# Residency and Spatial Use by Reef Sharks of an Isolated Seamount and Its Implications for Conservation

**DOI:** 10.1371/journal.pone.0036574

**Published:** 2012-05-16

**Authors:** Adam Barnett, Kátya G. Abrantes, Jamie Seymour, Richard Fitzpatrick

**Affiliations:** 1 Fisheries, Aquaculture and Coasts Centre, Institute for Marine and Antarctic Studies, Hobart, Tasmania, Australia; 2 School of Marine and Tropical Biology, James Cook University, Cairns, Queensland, Australia; 3 Reef Channel, Townsville, Queensland, Australia; University of California Davis, United States of America

## Abstract

Although marine protected areas (MPAs) are a common conservation strategy, these areas are often designed with little prior knowledge of the spatial behaviour of the species they are designed to protect. Currently, the Coral Sea area and its seamounts (north-east Australia) are under review to determine if MPAs are warranted. The protection of sharks at these seamounts should be an integral component of conservation plans. Therefore, knowledge on the spatial ecology of sharks at the Coral Sea seamounts is essential for the appropriate implementation of management and conservation plans. Acoustic telemetry was used to determine residency, site fidelity and spatial use of three shark species at Osprey Reef: whitetip reef sharks *Triaenodon obesus*, grey reef sharks *Carcharhinus amblyrhynchos* and silvertip sharks *Carcharhinus albimarginatus*. Most individuals showed year round residency at Osprey Reef, although five of the 49 individuals tagged moved to the neighbouring Shark Reef (∼14 km away) and one grey reef shark completed a round trip of ∼250 km to the Great Barrier Reef. Additionally, individuals of white tip and grey reef sharks showed strong site fidelity to the areas they were tagged, and there was low spatial overlap between groups of sharks tagged at different locations. Spatial use at Osprey Reef by adult sharks is generally restricted to the north-west corner. The high residency and limited spatial use of Osprey Reef suggests that reef sharks would be highly vulnerable to targeted fishing pressure and that MPAs incorporating no-take of sharks would be effective in protecting reef shark populations at Osprey and Shark Reef.

## Introduction

Many marine apex predators are under threat from direct exploitation, mortality as bycatch, competition with fisheries and from other anthropogenic impacts such as habitat alteration or degradation [1]–[7]. Since many predators are important for maintaining the stability and functional structure of ecosystems (e.g. by regulating mesopredator populations), the protection of these species is also effectively contributing to protecting ecosystem health and biodiversity [3], [8], [9]. However, the vulnerability of many apex predators, as a result of their K-selected life-history strategies generally characterized by low fecundity, slow growth and late age at maturity, hampers both their protection and attempts at rebuilding exploited populations, even after they have been protected [4], [10].

In general, total protection only occurs once a species is significantly reduced [11]. So, for vulnerable species that are not presently protected, other management methods are needed to preserve their populations. For species with a commercial value, this can be achieved through implementing quotas and size limits to the catch or temporal closures to the fishery. However, for species with little or no commercial value, other methods of protection are warranted. The formation of marine protected areas (MPAs) is a common method used to preserve both targeted fishery species and non targeted species alike [6], [12]–[14]. The protection of key habitats can also assist in facilitating population growth for some species. However, as protected areas are unlikely to encompass entire distribution ranges of large predator populations [6], [15], the challenge is to implement an area that is large enough to afford sufficient protection to species that are highly mobile, while also appeasing human activities [6], [16]. Therefore, defining important habitats within a species’ broader distribution range can reveal areas that are essential to a population’s survival [16]. For example, studies on killer whales *Orcinus orca* and African penguins *Spheniscus demersus* used behavioural information to prioritise habitats primarily used for the activity in which they are most receptive to anthropogenic disturbance [6], [16]. In both cases, the protection of essential foraging grounds was of greater benefit than protecting habitats generically.

Shark stocks are experiencing huge declines in numerous locations [10], [17]–[22], and with the high level of susceptibility this group has to over exploitation, there is a critical need for adequate conservation and management to protect their stocks [23]. The tropical Indo-Pacific Ocean area has experienced significant increases in the harvesting of shark species, driven by a growing demand for shark products from Asian markets [24], [25]. These heavy levels of exploitation in conjunction with habitat degradation are severely threatening shark populations in reef systems [25]. MPAs have been proposed for shark conservation [19]–[21], [26] but, to date, protected area management has mainly focused on life stages using coastal nursery areas [27]. Although MPAs are a common strategy implemented on coral reef systems to conserve predators, unfortunately these areas are often designed with little prior knowledge of the spatial behaviour of the species they are designed to protect [28], rendering MPAs ineffective if they fail to encompass a large part of the species’ home range [12], [21], [29]. Therefore, knowledge of the spatial ecology of sharks is essential for their appropriate management and conservation [7], [12], [27].

The Coral Sea region, an area of approximately 972 000 km^2^_,_ extends east of the Great Barrier Reef Marine Park (GBRMP) to the edge of Australia’s Exclusive Economic Zone. In May 2009, the entire Coral Sea region was declared a Conservation Zone to provide interim protection while the area is being assessed for potential inclusion in the Commonwealth Marine Reserves. Activities taking place in the Coral Sea region, including tourism and commercial and recreational fishing, were allowed to continue. Commercial fishing activities are currently licensed and managed through the Australian Fisheries Management Authority (AFMA) and include long lining, deep water trapping, sea cucumber collecting and aquarium fish collecting. These activities have an estimated value of ∼ $AUS1 million per annum [30], [31]. Charter vessel companies that offer a mix of rod and line and spear fishing provide the majority of recreational fishing. Tourism activities consisting of live-aboard dive vessels operating out of a number of ports in Queensland also operate in the area. Since September 2008, there has been a campaign in the Australian community to have the Coral Sea declared a Marine Park. The initial proposal is for a multiple use marine park model for the whole region that promotes sustainable use, similar to that of the Great Barrier Reef Marine Park (http://www.environment.gov.au/coasts/mbp/coralsea/publications/pubs/coralsea-reserve-proposal.pdf). Conservation groups and some scientists have proposed a total no-take model. To date, this no-take proposal has polarized the debate amongst the community and stakeholders. If MPAs are approved, the protection of sharks at Coral Sea seamounts should be an integral component of this planning. Therefore, knowledge on the spatial ecology of sharks at the Coral Sea seamounts is essential for the appropriate implementation of management and conservation plans.

In the initial proposed multi-use model, our study location, Osprey Reef (a seamount) is listed as a Habitat Protection Zone that would allow limited commercial fishing (handline/rod and reel, hand collection for the aquarium and sea cucumber trade) and recreational fishing. As with other isolated atolls and seamounts in the Indo-Pacific region, the shark assemblage at Osprey Reef is dominated by a few species [32], [33]. Grey reef sharks *Carcharhinus amblyrhynchos*, whitetip reef sharks *Triaenodon obesus* and silvertip sharks *Carcharhinus albimarginatus* are the most common species observed [34]–[36]. All three species are widely distributed across the Indo-Pacific, and whitetip and grey reef sharks are the most abundant shark species on many coral reefs [37]. However, due to their slow growth and low fecundity, all three species are believed to be vulnerable to exploitation and there is some evidence of population declines over parts of their distribution range [20], [37]–[42]. It is estimated that each year, live-aboard dive boats are directly responsible for generating at least AU$16 M worth of income to the Cairns/Port Douglas region (North Queensland) [34]. Of all the Coral Sea reef systems, Osprey Reef has the highest visitation rate by tourism operators, primarily to conduct shark dives [34]. So, the depletion of reef sharks at Osprey Reef would have financial ramifications for tourism in North Queensland. To put this into perspective, in the Maldives, the removal of only 20 grey reef sharks, with a market value of only AU$1 000, caused an estimated loss of AU$500 000 annually in diving revenue [43].

The objective of this study was to examine the patterns of spatial use of reef shark species to determine if an MPA would be an appropriate management strategy for protecting shark populations at Osprey Reef and the neighbouring Shark Reef. The specific aims were 1) determine if reef sharks are permanent residents around Osprey and Shark Reef, 2) investigate the spatial use of sharks at Osprey Reef and 3) examine inter- and intra-specific spatial overlap of sharks at Osprey and Shark Reefs.

## Methods

### Ethics Statement

All research methods were approved and conducted under Australian Fisheries Management Authority Scientific Permit #901193.

### Study Area and Acoustic Array Design

**Figure 1:**
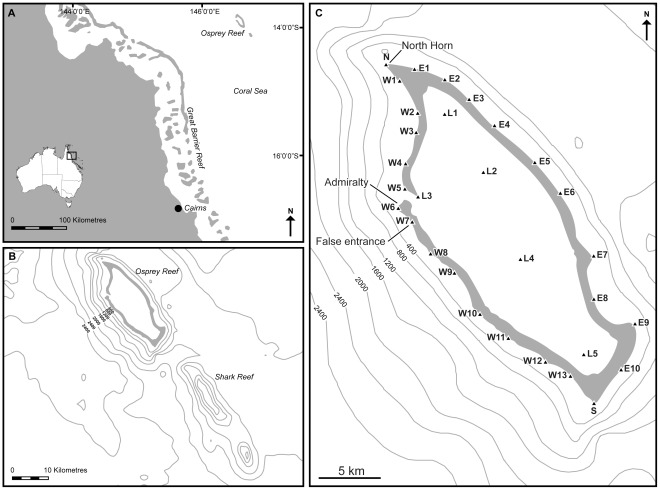
Location of Osprey Reef in the Coral Sea, Australia (A and B). Panel C shows the depth contours (in metres) around Osprey Reef, the VR2 receiver array forming a ring around the perimeter of Osprey Reef, and the 5 receivers within the lagoon. Triangles represent receivers. North Horn, Admiralty and False Entrance are the shark tagging locations.

Osprey Reef is an isolated seamount in the Coral Sea (13°54.190’S, 146°38.985’E), approximately 220 km east off the north-east coast of Australia, and 125 km from the edge of the Great Barrier Reef (Fig. 1). Osprey Reef rises vertically from 2400 m to just below the sea surface (up to less than 1 m), and is 25 km in length and 12 km wide, covering an area of about 195 km^2^ (Fig. 1). The east wall and southern end of the reef are exposed to the prevailing south-east winds, and the north-west corner is the least exposed area. The centre of the reef is a lagoon with a maximum depth of 40 m, characterised by sandy substrate scattered with coral bommies throughout. There is currently no commercial fishery targeting sharks at Osprey Reef. The only human influences in the area are from a sea cucumber fishery (hand collection), live aboard dive operators that often conduct shark feeds (see [36] for description of dive operations), a small amount of recreational fishing and minimal impact from aquarium collectors.

An array of 31 VR2w acoustic receivers (VEMCO Ltd., Halifax, Canada) was deployed from March 2008 to June 2009. Receivers were attached to and suspended above the reef with stainless steel chains, shackles and buoys. The array was configured to be non-overlapping, with 25 receivers forming a ring around the perimeter of Osprey Reef, and 5 receivers within the lagoon (Fig. 1). However, receivers W10 and W11 were lost due to anchorage failure resulting from a cyclone. An additional receiver was deployed at Shark Reef, a submerged atoll (top of reef 15 m, area ∼15 km^2^) ∼14 km south of Osprey Reef (Fig. 1). This is the only shallow structure in close proximity to Osprey Reef. The Shark Reef receiver was deployed to give some indication of the movements/connectivity between the two seamounts. Range testing of receivers showed that the distance at which 100% of transmissions were recorded was 400 m.

Sharks were tagged at four locations: North Horn, Admiralty and False Entrance, which are located on the outer edge of the reef and are popular dive sites (Fig. 1). The fourth location was in the lagoon, where only juvenile grey reef sharks were caught and tagged. No juvenile grey reef sharks were tagged at the other sites because none were observed. Although shark tagging was attempted at the east wall and southern Osprey, no animals were attracted to the bait or observed in the area from the boat or SCUBA diving.

### Study Species and Transmitter Attachment

Animals were fitted with acoustic-coded V16 4H transmitters (54 mm length×15 mm diameter; weight in water: 11 g; transmission off times: random between 50–150 s; battery life ∼3 years) (VEMCO Ltd., Halifax, Canada). Sharks were tagged between 25^th^ March and the 13^th^ April 2008, after all receivers had been deployed. In total, 18 adult whitetip reef sharks (112–150 cm TL), 27 grey reef sharks (16 adults, 9 juveniles, 2 sub-adults; 80–182 cm TL) and 4 adult silvertip sharks (157–230 cm TL) were tagged. With the exception of three grey reefs and one whitetip, all sharks were females.

Due to differences in behaviour and size, the three species were captured using different techniques. For whitetip reef sharks, animals were attracted to a closed crate containing fish frames (heads and skeletons) and secured to a barren reef outcrop. Sharks were then caught by a SCUBA diver gently grabbing the tip of the tail and quickly transferring the noose of a rope around the tail [36]. Sharks normally struggled for up to a minute before they relaxed and hang upside down from the rope. The diver then slowly surfaced and the sharks were brought aboard the boat for measurement and tag attachment (see [36]). Individuals were positioned on the duckboard of the boat and running water was pumped over the gills. Acoustic tags were implanted into the peritoneal cavity via a 1–2 cm incision in the abdominal wall, and the incision closed with surgical needle and absorbable thread. Sharks were returned to the water within 5 minutes.

Grey reef sharks were caught by handline using circle hooks or using a hook-less method. In the hook-less technique, sharks were attracted using a large tuna head threaded onto a stainless steel chain attached to a rope. The tuna head was thrown from a 6×2 m hydraulic platform on the back of the research vessel into the water and quickly pulled back in again. When a grey reef shark pursued the bait onto the platform, this was quickly raised thereby stranding the shark. Acoustic tags were implanted as described for whitetip reef sharks.

For silvertip sharks, two individuals were caught by hook and line, and internally tagged on the hydraulic platform of the boat as described for whitetip reef sharks. The other two individuals were tagged underwater. The transmitters were fitted with stainless steel tag heads and fitted to a modified spear gun. The sharks were attracted using the baitbox and a scuba diver shot the transmitter into the dorsal region of the shark.

### Data Analysis

The number of days that each individual was detected at Osprey and Shark Reef was plotted on a timeline to determine if individuals are permanent residents at this seamount. Other data analyses were based on the number of hours each shark was present at each area (i.e. each receiver), and this was used as an indication of how often an individual and a species used that area of the reef. If a shark was detected by a receiver more than once in any particular hour, it was considered as having been present during that hour.

The spatial overlap of the three species (for grey reef sharks, only adults were used in the analysis) was compared using niche overlap analysis conducted in EcoSim 700 [44], using Piankas index (*O*), permutated 1000 times. The degree of overlap is presented in a 0–1 scale, where 0 equals no overlap and 1 equals complete overlap. To complement the spatial overlap results, the proportion of hours each species was recorded at each receiver is graphically presented. Further spatial overlap analyses were also performed within species. For whitetip reef sharks, spatial overlap was tested between sharks tagged at three locations (North Horn, Admiralty and False Entrance), and for grey reef sharks spatial overlap was run to compare adults tagged at two locations (North Horn and Admiralty) and between life stages (adults from both locations vs. juveniles).

For whitetip and grey reef sharks, circular statistics (Oriana 3 software) were also used to study the diurnal pattern of area use around receivers that had the highest number of hits (hereafter termed as key locations): N, W1, W6, W7 and, for Grey Reef sharks, also W2 (Fig. 1). For each receiver, Rao’s Spacing Test (U) was used to test for uniformity in the temporal distribution of the detection data. Silvertip sharks were not included in this analysis because only four individuals were tagged, and two of which were not detected for much of the study period. For these analyses, the response variable was the number of individuals detected by a receiver at each of the 24 hours of the day, and replicates were the different days. Again, sharks were considered present only when detected by a receiver more than once at a particular hour. Sunrise time ranged from 05:50 h in summer to 06:40 h in winter, and sunset was between 18:10 h in winter and 18:55 h in summer.

A Fast Fourier Transform (FFT) was also computed for each species to identify any temporal periodicity in shark activity around receivers N and W6, as these had the highest number of detections for all species. Only adult individuals that were regularly detected at either receiver throughout the course of the study were considered in this analysis (whitetip reef sharks: *n* = 15, grey reef sharks: *n = *16, silvertip sharks: *n = 2*). Input data were the number of detections per hour blocks. A FFT separates time-series data into frequencies and identifies any sinusoid patterns, or periodicity, in the dataset. A power spectrum is then constructed and the dominant frequencies are represented by peaks in the spectrum [34]. Before analysis, data were smoothed with a Hamming window, a weighted moving average transformation used to smooth the periodogram values [45]. Windowing reduces discontinuity between frames, smoothes the data and reduces noise, thus improving the “quality” of the harmonics so that spectral leakage is reduced and it is easier to identify the frequencies that contribute the most for the overall periodicity of the time series. Time series analysis was done using Statistica v.7.0. (Statsoft, USA).

## Results

### Residency

**Figure 2:**
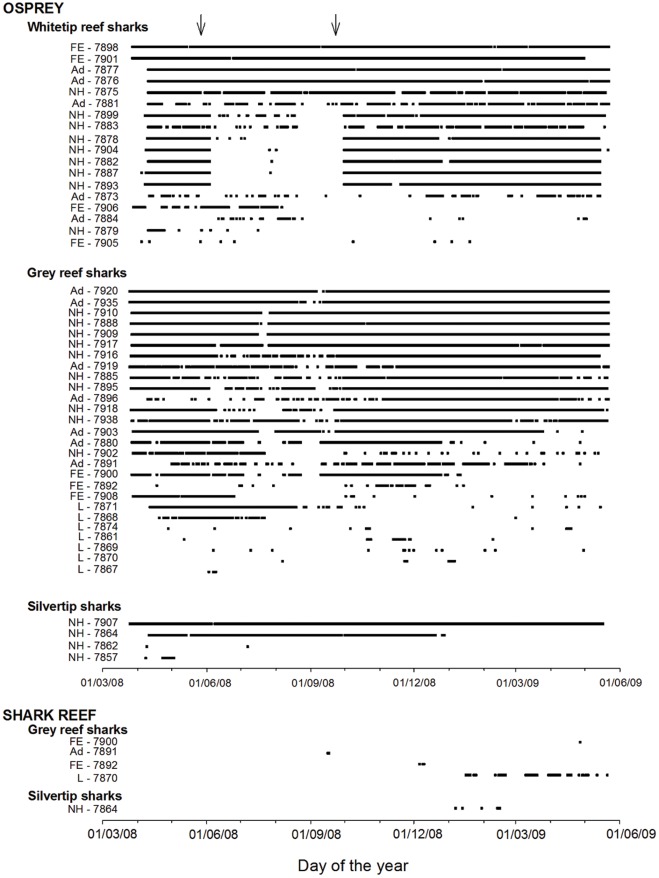
Timeline of the daily detections of acoustic coded individual sharks at Osprey Reef from March 2008 to June 2009. Individuals are classified by their tagging location (FE- False Entrance, Ad- Admiralty, NH – North Horn, L – lagoon) and acoustic transmitter ID. Note that all grey reef sharks tagged in the lagoon were juveniles. The arrows at the top of the graph represent the period from the 4^th^ June to the 29^th^ September when the North Horn (N) receiver had a battery failure.

Most animals were detected at Osprey Reef from the day of tagging until the end of the study (Fig. 2). The only exceptions were three silvertips, three whitetips and four juvenile grey reef sharks, for which detections stopped shortly (i.e. ∼5 weeks) after tagging (one silvertip), or animals were only detected on a few days throughout the study (the nine other individuals) (Fig. 2). There is a noticeable gap in the timeline, most evident for the whitetips tagged in North Horn, which suddenly stopped being detected from the 4^th^ June, with regular detections starting again on the 29^th^ September (Fig. 2). This was due to a battery failure at the North Horn receiver. The pattern is not so obvious for grey reef sharks because they move through wider areas, and were regularly within range of the adjacent W1 and E1 receivers, and therefore were still detected in the area each day. In contrast, whitetip reef sharks tagged at North Horn rarely left the vicinity of the area (see Discussion). However, outside of the battery failure period, whitetip reef sharks were detected almost every day (Fig. 2). The fact that all the North Horn whitetip reef sharks stopped being detected on precisely the same day and then resumed detections again on a precise day suggests they did not migrate out of the North Horn area. For both adult whitetip and grey reef sharks, 11 out of the 18 tagged adult individuals were detected on >90% of days after tagging. Because of the failure of the North Horn receiver, for sharks tagged at North Horn the days of receiver failure were not considered in this analysis.

### Spatial Use

**Figure 3:**
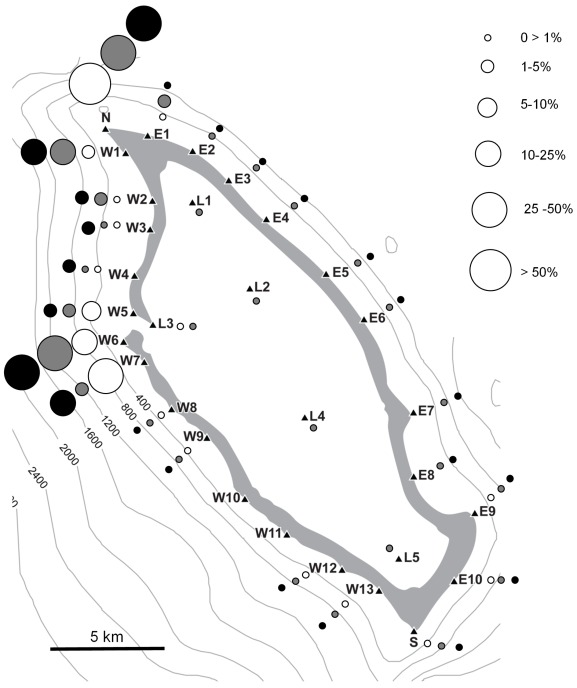
Bubble plot showing the percentage of hours that each species was detected at each receiver. White circles - whitetip reef sharks; grey circles - grey reef sharks; black circles - silvertip sharks.

**Figure 4:**
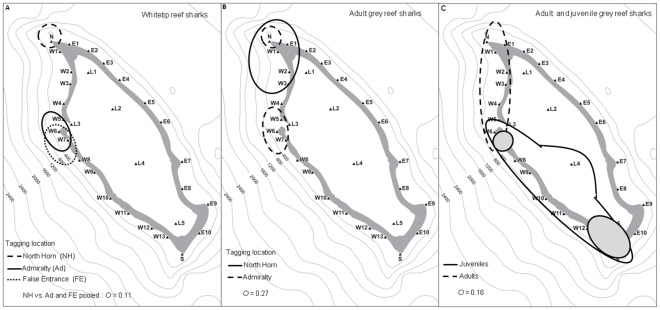
Area use maps, showing the 95% contours of the number of hours of detection occurred for sharks at different tagging locations and the comparison of spatial use with Pianka’s niche overlap value (*O*), where 0 represents no overlap and 1 equals total overlap. Panel A - whitetip reef sharks tagged at three locations.Note that for the *O* value calculation, sharks tagged at Admiralty and False Entrance were pooled and compared with North Horn individuals. Panel B – Adult grey reef sharks tagged at two locations. Panel C – All adult grey reef sharks compared with juveniles. The grey shaded area represents the 80% contours of juvenile hourly detections.

There was a strong spatial overlap in the area use by the three species (Pianka’s *O* = 0.85), in particular between grey reef and silvertip sharks (*O* = 0.98). All three species spent the majority of time at N, W6 and W7 locations (Fig. 3). Overall, the north-west corner of Osprey Reef was used far more than any other area, and the east wall and southern ends were rarely visited. In fact, whitetip reef sharks were never detected on the east wall and they didn’t move into the lagoonal area past receiver L3 (Fig. 3). The lagoon was only regularly used by the juvenile grey reef sharks (Fig. 4).

Five individuals, four grey reef and one silvertip sharks were detected at the single receiver at Shark Reef. Surprisingly, three of the grey reef sharks were juveniles (80–107 cm TL). One grey reef (7892) detected at W6 (Admiralty) on the 2^nd^ December 2008 moved to Shark Reef in four days, stayed at Shark Reef for at least four days and was detected back at Osprey by receiver W13 two days later. Another individual (7891) was detected by receiver SR on the 16^th^ of September 2008, 11.5 h after being within range of receiver S. It stayed in Shark Reef for at least one day, eight days later it was detected back at Admiralty, and remained in the Osprey area for the rest of the study. A third grey reef (7900) was detected at Shark Reef on the 27^th^ of March 2009, after a last detection at North Horn 41 days earlier. This individual was not recorded after this last detection at Shark Reef. The fourth grey reef (7870) moved from receiver S to Shark Reef in one day, on the 15^th^ January 2009, and remained in Shark Reef for the rest of the study. The silvertip shark (7864) was detected at Shark Reef on the 7^th^ January 2009, after a last detection in Admiralty on the 28^th^ December 2008. It was then within range of receiver SR until the 15^th^ February 2009, after which there were no more detections.

Whitetip reef sharks remained very close (i.e. within 5 km) to their tagging location for the duration of the study (Fig. 4a). A high overlap was evident for sharks tagged at Admiralty and False Entrance (*O* = 0.91; Fig. 4a). However, individuals tagged at North Horn showed extremely low spatial overlap with individuals tagged at Admiralty (*O* = 0.03) and False Entrance (*O* = 0.01) (North Horn vs. Admiralty and False Entrance pooled: *O* = 0.11) (Fig. 4). Adult grey reef sharks tagged at North Horn and Admiralty also showed a low degree of spatial overlap (*O* = 0.27), and adult and juveniles were even more spatially separated (*O* = 0.16; Fig 4b, c).

### Diel Patterns at Key Locations

**Figure 5:**
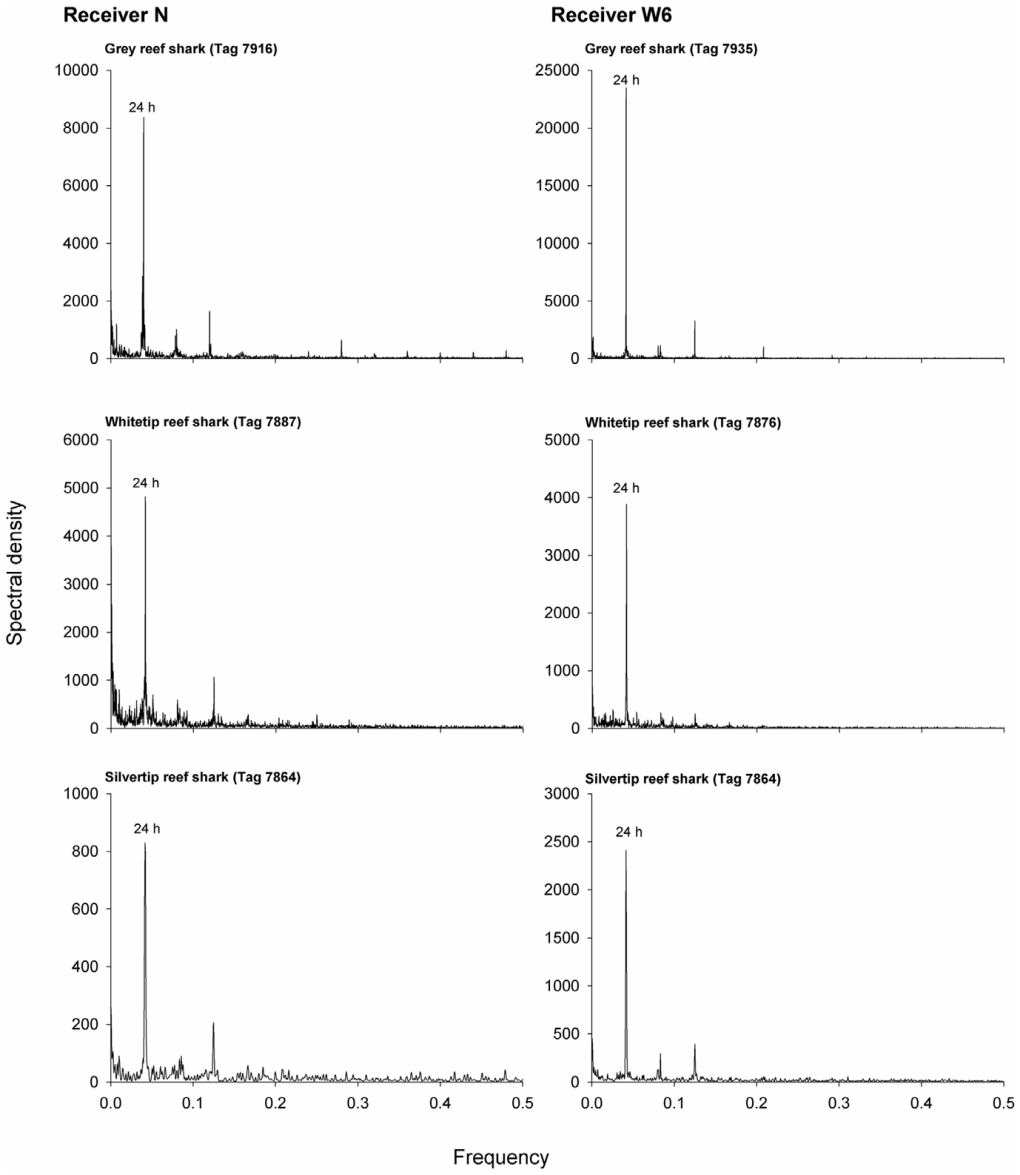
Fast Fourier transform of the time series of number of detections per hour for one representative individual of each species at receivers N and W6. Periodicities of peaks are given over the peaks. Receivers N and W6 were chosen for this analysis because these correspond to the tagging areas, and most individuals spent a large part of their time in the vicinity of the tagging place throughout the study. Therefore, data from these receivers provides more complete information on the dial activity periodicity. FFT analysis for the other adult individuals of the three species that were regularly detected at these receivers throughout the course of the study led to similar results.

**Figure 6:**
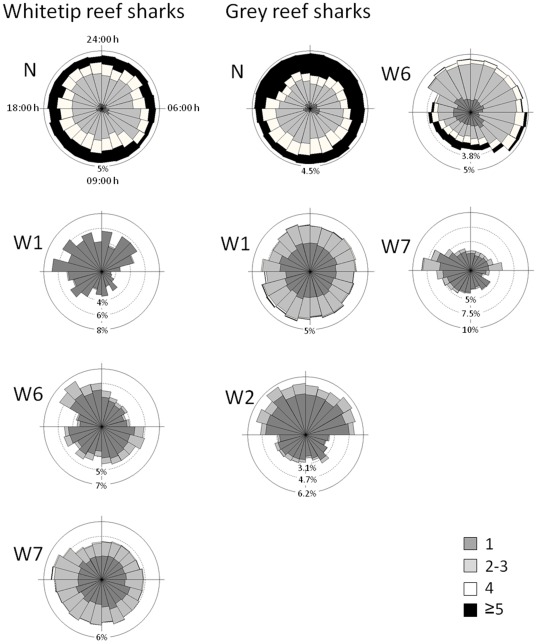
Circular plots showing the distribution of the number of individuals detected at each hour of the day for each of the main receivers (in % of total) for whitetip and grey reef sharks. Note the differences in scale between plots. Different shadings represent the number of sharks detected in a given hour.

A circadian (24 h) periodicity in the use of receivers N and W6 was present for all adults of the three species that were frequently detected at these sites (Fig. 5). For whitetip reef sharks tagged in North Horn, the diurnal pattern is somewhat hard to detect visually with the circular graphs (Fig. 6), as whitetips spent the majority of their time in the vicinity of receiver N. The distribution of number of sharks detected per hour was however non-homogeneous (Rao’s spacing test, p<0.01), driven by more whitetips being detected per hour during the day, and less individuals detected in the area around dawn, i.e. between 6:00 and 8:00 h (Fig. 6). Also, more whitetips occurred during the night at receiver W1 (Fig. 6), the closest to receiver N (Rao’s test, *p*<0.01). Whitetips tagged in Admiralty and False Entrance spent most of their time at receivers W6 and W7 (Fig. 3). These animals showed a non-uniform (Rao’s test, *p*<0.01) bimodal pattern in the use of W6, with more individuals being detected in the first hours of the night and in the first hours of the day (Fig. 6). At W7, whitetip reef shark occurrence was higher during the day before peaking at dusk to early evening, between 18:00 and 21:00 h (Rao’s test, *p*<0.01) (Fig. 6).

Grey reef sharks tagged at North Horn showed diel difference in the use of receivers N, W1 and W2 (Rao’s test, *p*<0.01) (Fig. 6). The pattern suggests that they expand their area use at night, moving down the reef from North Horn to W2 (Fig. 6). Grey reef sharks tagged at Admiralty used the W7 area less during the day (Rao’s test, *p*<0.01) (Fig. 6), and then peaked in activity around dusk at W7 (Rao’s test, *p*<0.01) (Fig. 6).

## Discussion

Results suggest that reef sharks at Osprey Reef are permanent residents of the area (Osprey and neighbouring Shark Reef), with very little emigration away from these areas. It is however important to note that not all animals were detected for the entire study period, and this needs to be considered when evaluating emigration and possible immigration of sharks to and from the Osprey population. There are a number of possible reasons why some animals (6 adult and 4 juvenile sharks) were not detected for the entire study: animals could still be present but were in areas of low receiver coverage, they could have emigrated from Osprey Reef, could have died during the study, or the transmitters failed. All four reasons are plausible, and it could well be a combination of them. As the results of whitetip reef shark movements suggest this species has small centres of activity, the three individuals that were not detected for the duration of the study could have moved to an area with no receiver coverage. For example, two of these individuals were tagged at False Entrance (W7) and they could have moved further south into the area where the two receivers were lost (W10 and W11). Also, there was only one receiver at Shark Reef, so animals could have moved there without being detected. Indeed, individuals moving to and from Shark Reef went days without being detected (11.5 hours to 41 days). For the four juvenile grey reef sharks, mortality could be responsible for the cessation of detections, as juvenile sharks are vulnerable to natural mortality by predation and starvation [46]–[48]. Alternatively, given that three juveniles moved to Shark Reef, they may have spent considerable time in that area but were not detected. Emigration of approximately 134 km to the Great Barrier Reef (GBR) for adult grey reef and silvertip sharks is also a possibility since shark 7908 was detected at the GBR 47.8 h after its last detection at Osprey Reef on the 25^th^ June 2008 [49] and was subsequently detected back at Osprey Reef on the 2^nd^ of October 2008. Finally, the two silvertip sharks for which detections stopped during the study were those that were externally tagged, so this could be a result of the tags being removed or falling off the sharks. Although the expulsion of internally implanted tags has been recorded in teleosts [50], to date, no tag loss has been documented from acoustic tags implanted in the body cavity of sharks.

An interesting aspect of the population structure of whitetip and grey reef sharks at Osprey Reef is the sex ratio, which appears to be strongly biased towards females (pers. obs. of authors, with >12 years of frequent diving at Osprey Reef). This was reflected in the low number of males tagged in the current study (one whitetip and three grey reefs). So, do the few males mate with all females in the area, or is there a temporary emigration by females (as seen by shark 7908) or immigration of males from other areas? In Hawaii, female whitetip reef sharks show higher philopatry than males, which may suggest that males move more than females [42]. However, an Indo-Pacific study based on genetics suggests that whitetip reef sharks are site attached, with sharks from two regions of the GBR displaying unprecedented genetic isolation for a carcharhinid species [51]. At Osprey reef, mating scars on females of both species are evident during the summer periods, between November and December, and newborn whitetips have been observed at North Horn hiding amongst the reef during the summer months (authors’ pers. obs.). Quantitative studies incorporating genetics and population structure and abundance (e.g. photo ID for mark and recapture studies) are required to further explore residency and the population dynamics of both species at Osprey and Shark Reefs and the consequences that biased sex ratios may have to the effectiveness of any implemented MPA.

For whitetip reef sharks, the small area use reported at Osprey Reef resulted in very low overlap or mixing between sharks residing in different areas separated by only ∼10 km. This agrees with previous estimates that their movements are limited to 3–5 km [42], [52]. However, movements of up ∼26.4 km (straight line) have also been recorded for some individuals in Hawaiian reefs [42]. These larger movements have been interpreted as representing home range shifts [42]. In the present study, whitetip reef sharks from North Horn expanded their area coverage from the centre of activity at North Horn down the west wall to the area around receiver W1 at night. Whitetips at North Horn also increase vertical activity at night [36]. Both results confirm that whitetip reef sharks at Osprey Reef are nocturnally active, maintaining a central location or core area to rest during the day, before dispersing at night. Resting in a core area during the day and dispersing at night to likely forage has been reported for a number of shark species [27], [53], including grey reef sharks [12], [54].

Grey reef sharks at North Horn showed diel patterns of activity similar to whitetip reef sharks, but over a greater area. For example, during the day their centre of activity was around North Horn and W1, but at night they moved further down the west wall to the area around receiver W2 (see Fig. 6). The lower number of sharks detected at North Horn and W6 during the night hours could also be influenced by grey reef sharks leaving the proximity of the reef at night to forage in the pelagic zone, out of receiver range. Nevertheless, the high use of the North Horn area by both whitetip and grey reef sharks over the entire diel cycle suggests limited dispersal, with resting, foraging and any possible social behaviours all occurring in close proximity to North Horn. High spatial overlap between these two species also occurred at a coastal aggregation site in Ningaloo Reef, Western Australia [54].

The predictable use of particular sites by reef sharks suggests that determining the ecological significance of these sites is crucial for conservation planning. For example, whitetip and grey reef sharks showed peaks of activity in the use of the W7 area around the dusk period. False Entrance (W7) is an area where the reef wall is broken up, with many gullies and crevices (pers. obs). This may make it a good area for foraging at dusk, when reef fish are in the transitional stage from diurnal to nocturnal behaviour. Crepuscular peaks in activity have been observed for both captive and wild whitetip reef sharks (Whitney et al., 2007; [36], [55]. On the other hand, the high use of the W6 area may be influenced by the occurrence of a major cleaning station in the area, of which both silvertip and grey reef sharks are regular visitors [35]. However, the regular use of this area suggests that it is important for other reasons such as foraging. This area is located at a point of the reef with a ridge at 28 m and a steep drop off to 700 m [35], with strong tidal flows from water entering and exiting the lagoon. These are habitat features that grey reef sharks have previously been associated with [56], [57]. The repeated use of specific sites within Osprey Reef, a known hotspot for sharks, is similar to the spatial use of scalloped hammerhead sharks *Sphyrna lewini* in the Galapagos Islands, where sharks mainly use a few key areas around the Wolf Island hotspot [58].

The low spatial overlap between grey reef shark adults tagged 10 km apart was somewhat surprising, given that an individual moved to the GBR and returned within 4 months, and grey reef sharks display little site fidelity to reefs on the GBR [49]. Recent studies have reported similar low spatial overlap and strong site fidelity for large mobile sharks over relatively small spatial scales (i.e. 10s km) [59], [60], and grey reef sharks at an isolated group of atolls 250 km off the north west coast of Australia (Rowley Shoals) also appeared to show strong site fidelity to relatively small areas [12]. However, the actual area use or dispersal patterns of individuals could not be established because of limited receiver coverage over relatively small sections of large reefs [12]. In contrast to the Rowley Shoals study, in the present study the Osprey seamount had broader receiver coverage and therefore afforded a much more comprehensive view of area use for grey reef sharks. Essentially, results from the current study confirm the earlier predictions of Field et al. [12] at the Rowley Shoals, that grey reef sharks are site-specific within isolated atolls/seamounts. Strong site fidelity in grey reef sharks has also been recorded at Enewetak Atoll [61]. The differing movement patterns observed at oceanic atolls/seamounts and the GBR probably reflect the spatial context of the tagging studies. The GBR system consists of approximately 2300 Km of relatively closely-spaced reefs, where groups of reefs can be considered continuous habitat for grey reef sharks to move between. This is in contrast to the isolation of the deep sea atolls/seamounts [12], [49].

The dissimilarity in movement patterns between grey reef sharks on the GBR and at Osprey Reef highlights the dissimilar behaviour that can be observed for the same species in different locations. It also shows the uniqueness of different systems when considering protection plans. The conservation plan currently implemented in the GBR is considered to have limited utility for grey reef shark protection [49], in contrast, due to the long-term residency and strong site attachment of grey reef sharks at Osprey and Shark Reefs, the implementation of a marine protected area that incorporates no-take zones for sharks in this area could be effective for this species.

Due to their isolation, oceanic seamounts can still constitute relatively pristine functioning systems that support a diverse array of species [18]. Therefore, many are ideal candidates for MPAs because a large proportion of the animals living at these places will spend their entire lives within the protected zone. This ideology appears to be well suited to the reef shark populations at Osprey Reef. Despite the possible emigration of a few animals, whitetip and grey reef sharks at Osprey are highly site attached, displaying predictable spatial use patterns, and are therefore vulnerable to exploitation. This vulnerability is further evidenced by the low inter-species spatial overlap and the strong dependence on N, W6 and W7 areas, meaning that residents in a small area could be easily targeted. Also, the isolation of Osprey Reef suggests immigration would be low and, consequently, re-stocking ability of reef shark species after exploitation could be limited. In addition, the small spatial use exhibited by sharks at North Horn suggests they interact with ecotourism shark provisioning activities year round, see [36] for a description of tourism and shark interactions. Possible implications are 1) sharks in this area are easily attracted to boats as they are already conditioned to handouts [36], and 2) constant exposure to tourism activities can affect long-term behaviour and health [36], [62], [63].

Overall, there is a lack of information regarding the spatial use of large mobile species, and this makes it difficult to provide technical advice on the design and spacing of MPAs [58]. However, recent studies have evaluated the effectiveness of MPAs for protecting shark populations, e.g. [58], [64], but most were conducted after the MPAs have been implemented. The current study is one of the first studies to test the effectiveness of a MPA in protecting sharks prior to designing and implementing a marine reserve. If shark no-take zones are implemented at Osprey and Shark Reef then whitetip and grey reef shark populations should benefit from this protection. But, ideally, a protection zone should extend a significant distance from the reef to incorporate areas used by reef associated species such as silvertip and hammerhead sharks. However, further research is needed on these more mobile species to determine the appropriate size of a pelagic buffer zone. A similar protection plan was advised for the Galapagos Islands for scalloped hammerhead sharks, where appropriate protection might be provided by closing the areas where this species aggregates, and extending this protection outward to include the area covered during nocturnal foraging movements [58].

Few tropical marine systems have remained unaffected by human exploitation [65], so the depletion of apex predators often precedes the study and monitoring of coral reef systems [66], [67]. Consequently, protected areas are generally introduced after exploitation is well underway, making it difficult to establish the natural baseline for future studies [33]. Therefore, isolated coral reefs such as Osprey, which lack a history of intense exploitation, can be useful to evaluate human impacts, providing insights into the original ecological function of coral reef systems, and helping predict impacts of future exploitation (e.g. the comparison between communities from pristine and exploited reefs can give information on the likely consequences of future exploitation of pristine systems), and help devise strategies for protecting and re-building depleted predator populations in other regions, e.g. [18], [33], [67]. For example, studies at relatively pristine and exploited atolls in the northern Line Islands in the central Equatorial Pacific show that even modest fishing effort can drastically reduce apex predators and have negative effects on fish assemblage structure at coral reef atolls [18], [33]. Based on the information presented in the current study and from other isolated atoll/seamount locations [18], [33], [67], if the proposed Coral Sea Conservation Zone is implemented, it will provide a great deal of protection to shark species at Coral Sea seamounts and species that are susceptible to overfishing [21], [23].
